# Ir^0^/graphdiyne atomic interface for selective epoxidation

**DOI:** 10.1093/nsr/nwad156

**Published:** 2023-05-24

**Authors:** Zhiqiang Zheng, Lu Qi, Yaqi Gao, Xiaoyu Luan, Yurui Xue, Feng He, Yuliang Li

**Affiliations:** Shandong Provincial Key Laboratory for Science of Material Creation and Energy Conversion, Science Center for Material Creation and Energy Conversion, School of Chemistry and Chemical Engineering, Shandong University, Jinan 250100, China; Shandong Provincial Key Laboratory for Science of Material Creation and Energy Conversion, Science Center for Material Creation and Energy Conversion, School of Chemistry and Chemical Engineering, Shandong University, Jinan 250100, China; Shandong Provincial Key Laboratory for Science of Material Creation and Energy Conversion, Science Center for Material Creation and Energy Conversion, School of Chemistry and Chemical Engineering, Shandong University, Jinan 250100, China; Shandong Provincial Key Laboratory for Science of Material Creation and Energy Conversion, Science Center for Material Creation and Energy Conversion, School of Chemistry and Chemical Engineering, Shandong University, Jinan 250100, China; Shandong Provincial Key Laboratory for Science of Material Creation and Energy Conversion, Science Center for Material Creation and Energy Conversion, School of Chemistry and Chemical Engineering, Shandong University, Jinan 250100, China; CAS Key Laboratory of Organic Solids, Institute of Chemistry, Chinese Academy of Sciences, Beijing 100190, China; Shandong Provincial Key Laboratory for Science of Material Creation and Energy Conversion, Science Center for Material Creation and Energy Conversion, School of Chemistry and Chemical Engineering, Shandong University, Jinan 250100, China; CAS Key Laboratory of Organic Solids, Institute of Chemistry, Chinese Academy of Sciences, Beijing 100190, China; School of Chemical Sciences, University of Chinese Academy of Sciences, Beijing 100049, China

**Keywords:** graphdiyne, atom catalyst, atomic interface, selective epoxidation, high-performance conversion

## Abstract

The development of catalysts that can selectively and efficiently promote the alkene epoxidation at ambient temperatures and pressures is an important promising path to renewable synthesis of various chemical products. Here we report a new type of zerovalent atom catalysts comprised of zerovalent Ir atoms highly dispersed and anchored on graphdiyne (Ir^0^/GDY) wherein the Ir^0^ is stabilized by the incomplete charge transfer effect and the confined effect of GDY natural cavity. The Ir^0^/GDY can selectively and efficiently produce styrene oxides (SO) by electro-oxidizing styrene (ST) in aqueous solutions at ambient temperatures and pressures with high conversion efficiency of ∼100%, high SO selectivity of 85.5%, and high Faradaic efficiency (FE) of 55%. Experimental and density functional theory (DFT) calculation results show that the intrinsic activity and stability due to the incomplete charge transfer between Ir^0^ and GDY effectively promoted the electron exchange between the catalyst and reactant molecule, and realized the selective epoxidation of ST to SO. Studies of the reaction mechanism demonstrate that Ir^0^/GDY proceeds a distinctive pathway for highly selective and active alkene-to-epoxide conversion from the traditional processes. This work presents a new example of constructing zerovalent metal atoms within the GDY matrix toward selective electrocatalytic epoxidation.

## Teaser

An advanced catalytic system of graphdiyne-based zerovalent iridium atom catalysts with unique incomplete charge transfer between Ir^0^ atoms and graphdiyne was developed to maximize atomic efficiency for epoxidation of olefins in aqueous solutions.

## INTRODUCTION

Selective oxidation of olefins to epoxides is an important industrial process for producing valuable intermediates and fine chemicals, and has attracted increasing attention in both academic and industrial fields [1–4]. However, current olefin oxidation processes are generally operated at high temperatures and high pressures under the presence of catalysts and oxidants. Therefore, many serious issues are inevitably involved, such as undesirable side reactions, poor product selectivity, large amounts of CO_2_ emission, serious environmental pollution, and difficulty in recycling the catalysts [5–7]. Scientists have put in a lot of effort in order to better promote the development of the field, and many catalysts have been reported to improve the performance of the olefin oxidation processes [1–4]. However, the FE and selectivity to target products are still at very low levels, which are serious obstacles to realizing efficient catalysis and utilization of energy and resources.

The development of selective and efficient electrocatalysts is the only way to convert olefins to target products by the direct utilization of electricity and green water (as the oxygen source) at ambient conditions [6–9]. However, how to realize the electrocatalytic conversion of olefin-to-epoxide at high Faradaic efficiency (FE) has to overcome the following issues: (i) the inefficient water oxidation that cannot provide sufficient oxygen intermediates to increase the reaction rate; (ii) the prevention of uncontrolled over-oxidation of olefins to undesired products; (iii) the low conductivity of olefin/water system. What we need to do is to rationally design and prepare an efficient and transformative electrocatalyst to achieve efficient olefin-to-epoxide conversion with high selectivity and high epoxide yield.

Zerovalent AC, comprising of individual metal atoms highly dispersed and anchored on the surface of GDY, is one of the hottest research frontiers in the catalysis field and is considered as an ideal catalytic model system for the fundamental and conceptual study of electrocatalysis [10–42]. Remarkably, the incomplete charge transfer between individual metal atoms and GDY greatly improves the charge transfer ability of the system, enhances conductivity resulting in a large number of active sites and maximum atomic efficiency, and can realize the selective and fast transport of electrons, efficient activation of reactant molecules, and effective stabilization of reaction intermediates, therefore resulting in excellent reaction selectivity and intrinsic catalytic activity for versatile reactions [43–50]. Moreover, the well-defined chemical/electronic structures and determined valence states of ACs provide natural advantages for identifying the active sites and understanding the structure-performance relationship at the atomic level.

Herein, we report the synthesis of a new zerovalent Iridium atoms catalyst which is highly dispersed and anchored on the surface of GDY and directly electro-oxidizes ST to SO using water as the source of oxygen atoms. Detailed characterization results show that the Ir atoms are zerovalent and independently dispersed and anchored on the surface GDY. *In-situ* characterization of the olefin-to-epoxide conversion processes demonstrate that the Ir^0^/GDY can not only effectively catalyze water splitting to provide oxygen species, but also facilitate the exchange of electrons between the catalysts and the reactant molecules. By using this strategy, we achieved epoxide production at a high conversion efficiency of ∼100%, high SO selectivity of 85.5%, and high FE of over 55% at ambient temperatures and pressures. The reaction mechanism is elaborated via the combination of theoretical calculations and *in situ* spectroscopy characterizations.

## RESULTS AND DISCUSSION

**Figure 1.**
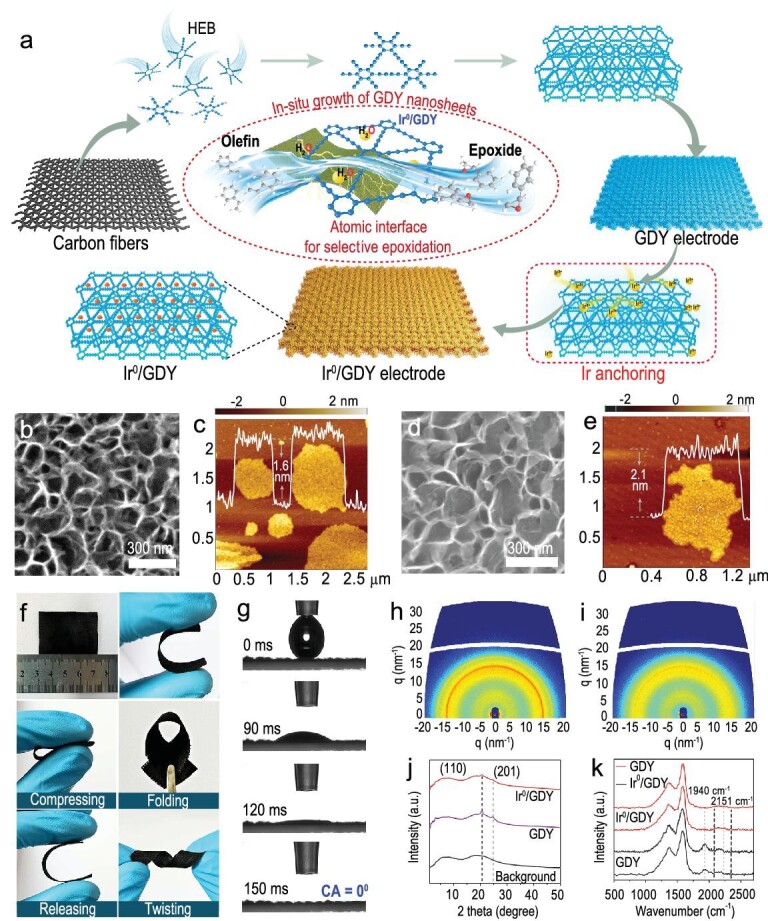
(a) Schematic illustration of the synthesis routes to Ir^0^/GDY. (b) SEM and (c) AFM images of GDY. (d) SEM and (e) AFM images of Ir^0^/GDY. (f) Photographs of Ir^0^/GDY electrodes. (g) Contact angle measurements on Ir^0^/GDY. 2D GIWAXS pattern of (h) GDY and (i) Ir^0^/GDY. (j) Diagonal plots from (h) and (i), numerical values denote Miller indices. (k) Raman spectra of GDY (black lines) and Ir^0^/GDY (red lines).

**Figure 2.**
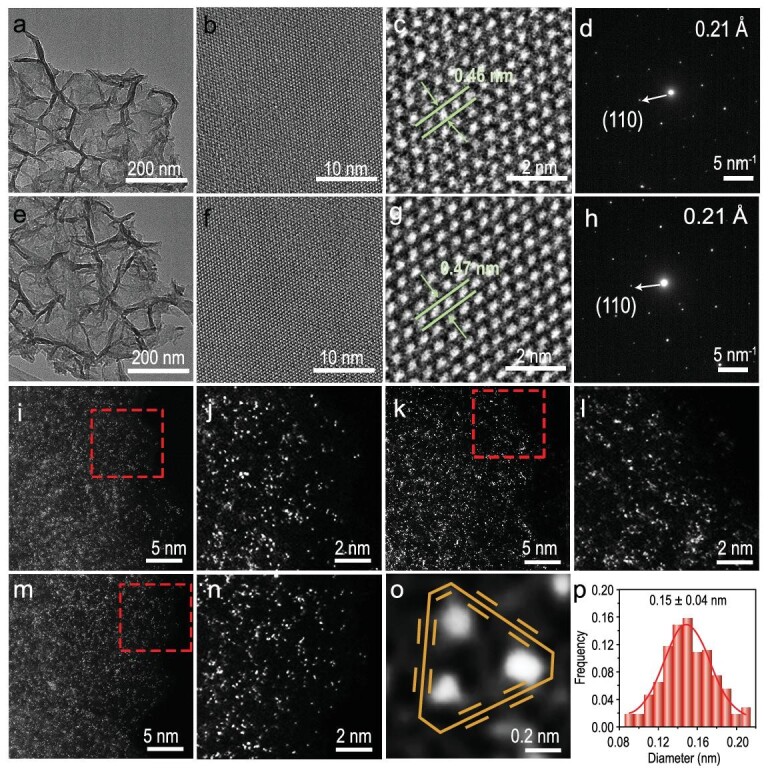
(a) TEM, (b and c) HRTEM, and (d) SAED images of GDY nanosheets. (e) TEM, (f and g) HRTEM, and (h) SAED images of Ir^0^/GDY. (i, k and m) HAADF-STEM images of Ir^0^/GDY from different samples. (j, l and n) Enlarged images of the marked areas in (i), (k) and (m), respectively. (o) Configurations of single Ir atoms anchored on GDY. (p) Size distribution of Ir atoms anchored on the surface of GDY.

Ir^0^/GDY was synthesized through a simple method (Fig. 1a) including the *in-situ* growth of a film of GDY nanosheets (SEM: Fig. 1b, Figs S1 and S2; AFM: Fig. 1c, ∼1.6 nm) on the smooth surface of carbon fiber (Fig. S3), followed by the anchoring of Ir^0^ on GDY. GDY nanosheets were controllably grown on the surface of carbon fibers through the Glaser–Hay coupling reaction of hexaethynylbenzene by using Cu as the catalysts, in an autoclave reactor at 110°C for 12 h [13]. For Ir^0^/GDY, the nanosheet morphology was well maintained (Fig. 1d, Fig. S4) but had a slight increase in thickness (∼2.1 nm, Fig. 1e) due to the anchoring of Ir (Fig. S5; mass loading: 3.06 wt%). Ir^0^/GDY exhibits high flexibility (Fig. 1f) and super-hydrophilic characteristics (Fig. 1g), which are helpful for practical uses. 2D grazing-incidence wide-angle X-ray scattering (GIWAXS) patterns (Fig. 1h; Fig. S6), transmission electron microscopy (TEM, Fig. 2a), high-resolution TEM (HRTEM, Fig. 2b and c) and selected area electron diffraction (SAED, Fig. 2d) results confirm the high crystallinity of GDY. Interestingly, the high crystallinity (Fig. 1h–j; Fig. 2e–h; Fig. S7) and structural integrity (Fig. 1k, Fig. S8a) of GDY could be maintained after the anchoring of zerovalent Ir atoms. Ir^0^/GDY exhibits the larger R_D/G_ (0.61) than that of GDY (0.58), which indicates the more defective sites formed after the anchoring of zerovalent Ir atoms on GDY. Compared with GDY (0.46 nm), the lattice spacing of Ir^0^/GDY increased to 0.47 nm due to the strong interactions between Ir atoms and GDY. C 1s XPS spectra (Fig. S8b) of GDY and Ir^0^/GDY show four peaks of sp^2^–C, sp–C, C–O and C=O species, with the same sp–C/sp^2^–C ratios of 2, indicating the integrity of GDY after Ir anchoring. The newly appeared π–π* transition peak at 290.1 eV for Ir^0^/GDY reflects the interactions between Ir and GDY. Compared with GDY, sp–C peak of Ir^0^/GDY shows a negative shift in binding energy, indicating the electron transfer from Ir atoms to GDY.

**Figure 3.**
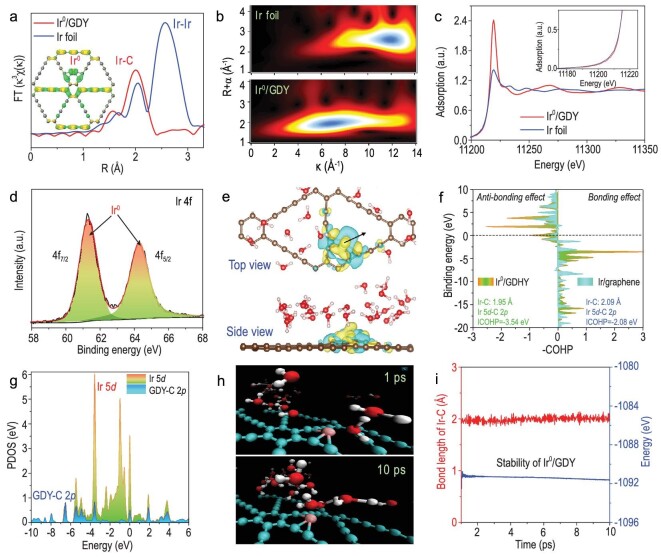
(a) Ir L_3_-edge EXAFS spectra of Ir^0^/GDY and Ir foil (inset: simulated model of Ir^0^/GDY). (b) WT for the k_3_-weighted EXAFS signals of Ir^0^/GDY and Ir foil with optimum resolutions of 2.0 Å. (c) Normalized Ir L_3_-edge XANES spectra of Ir^0^/GDY and Ir foil (inset in c: the enlargement of the marked area). (d) Ir 4f XPS spectra of Ir^0^/GDY. (e) Top and side views of charge-density difference (CDD) of Ir^0^/GDY. Isosurface is set to 0.003 e/bohr^3^. (f) COHP of the Ir-C bond in Ir^0^/GDY and Ir^0^/Graphene. (g) The PDOS of Ir 5d and C 2p in Ir^0^/GDY. (h) Molecular dynamics simulations of Ir^0^/GDY in water (10 ps). (i) AIMD results of Ir-C bond in Ir^0^/GDY.

High-angle annular dark field scanning transmission electron microscopy (HAADF-STEM) images show that bright dots (Ir atoms) were separately and uniformly anchored on GDY (Fig. 2i–n; Fig. S9). Theoretical simulations on the anchoring sites of single Ir atoms on GDY showed that Ir atoms favorably anchored at the corner of the acetylene rings with the largest binding energy than other sites on GDY (Fig. 2o, Fig. S10). The size distribution analysis (Fig. 2p; 0.15 ± 0.04 nm) confirms that Ir atoms exist as a single atom. Extended X-ray absorption fine structure (EXAFS) spectra (Fig. 3a; Fig. S11, Table S1) show that Ir^0^/GDY has a prominent peak at 2.01 Å corresponding to the Ir-C coordination rather than the Ir-Ir coordination (2.6 Å), in accordance with the wavelet transforms (WT) results (Fig. 3b). These results confirm individual Ir atoms were anchored on GDY in isolation. Ir L_3_ absorption edge of Ir^0^/GDY and Ir foil located at similar binding energies (Fig. 3c) indicate that Ir atoms in Ir^0^/GDY are metallic. The first derivative of the X-ray absorption near edge structure (XANES) spectra (Fig. S12) and Ir 4f XPS spectra (Fig. 3d) confirmed Ir atoms are zero-valent [51,52]. Charge density analysis shows that Ir atoms were anchored between the areas of electron accumulation and electron depletion (Fig. 3e) with reversible incomplete charge transfer between Ir and GDY. Crystal orbital Hamilton populations (COHP, Fig. 3f) show that the integrated COHP of Ir-C bond for Ir^0^/GDY (−3.54 eV) is larger than that of Ir-C bond for Ir/Graphene (i.e. −2.08 eV), indicating much stronger bonding and antibonding effects in Ir^0^/GDY than those in Ir/Graphene. Besides, the distance between Ir and C in Ir^0^/GDY (1.90 Å) is smaller than that in Ir/Graphene (2.09 Å), which reveal the stronger interactions between Ir and GDY than between Ir and Graphene. The projected density of states (PDOS) of Ir^0^/GDY show that Ir 5d orbitals were pinned around the Fermi Level by C 2p orbitals (Fig. 3g), which can effectively maintain the electron-rich state and high catalytic activity of Ir atoms during the catalytic reaction processes. Ab-initial molecular dynamics (AIMD) simulations show that Ir atoms can be stably anchored on the vertex of the triangular hole of GDY (Fig. 3h) within 10 ps. In addition, the bond length of Ir-C and the ground state energy of Ir^0^/GDY fluctuate over a very small range within 10 ps (Fig. 3i), confirming the excellent structural stability of Ir^0^/GDY. Electrochemical impedance spectroscopy (EIS; Fig. S13a) and electrochemical active surface area (ECSA) measurements (Fig. S13b) confirm the excellent conductivity and intrinsic activity of Ir^0^/GDY.

**Figure 4.**
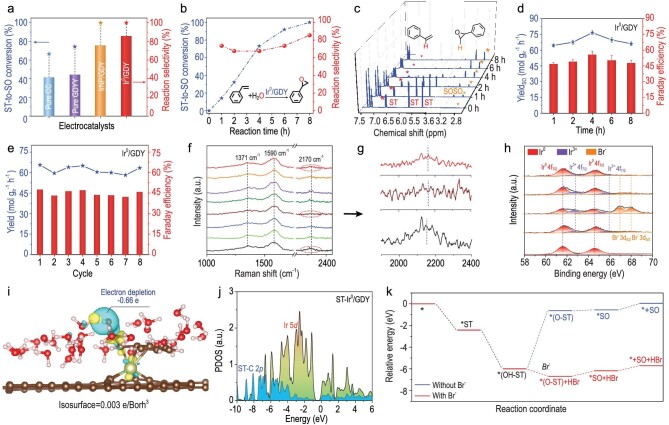
(a) Catalytic performances of the samples (reaction conditions: 0.25 M ST, 0.25 M NaBr, 2 mL electrolyte, 5 mA cm^−2^, 8 hour). (b) Time course of catalytic performances of Ir^0^/GDY. (c) ^1^H-NMR spectra recorded from the electrolytes. (d) Time course of the Y_SO_ and FE. (e) Cycling stability test. (f) *In-situ* Raman spectra of Ir^0^/GDY. (g) Enlargement of the marked areas in (f). (h) Ir 4f and Br 3d XPS spectra of Ir^0^/GDY during the electrocatalysis (fitted peaks: red line). (i) CDD of ST-adsorbed Ir^0^/GDY. Isosurface is set to 0.003 e/bohr^3^. (j) PDOS of Ir 5d and C 2p in ST-adsorbed Ir^0^/GDY. (k) Relative energy pathway of the electrocatalysis.

For electrochemical studies, the mass (Fig. S14) and ^1^H NMR (Figs S19–S28) spectra were used to confirm the identity of the products. As expected, Ir^0^/GDY showed better catalytic performance than reference samples (Figs S15–S22; Table S2), giving the highest ST-to-SO conversion (C_SO_) of 100% and reaction selectivity (S_SO_) of 85.5% (Fig. 4a) than IrNP/GDY (Fig. S22), CC (Fig. S19) and GDY (Fig. S20). With the increase in the reaction time, both the C_SO_ and the S_SO_ of Ir^0^/GDY increased (Figs S23–S28 and Table S3), as evidenced by the ^1^H-NMR spectra (Fig. 4c). The highest Y_SO_ and FE could reach 76.6 mol g_Ir_^−1^ h^−1^ and 55.3%, respectively (Fig. 4d; Tables S4–S6). Remarkably, such excellent catalytic performance of Ir^0^/GDY could be maintained for 64 hours (8 continuous cycles) without obvious decrease (Fig. 4e). HAADF-STEM (Fig. S29) and XPS (Figs S30 and S31) results confirmed the excellent long-term stability of Ir^0^/GDY.

*In-situ* Raman measurements, XPS analysis and DFT calculations were performed to gain deeper insights into the reaction mechanisms. As shown in Fig. 4f and g, the intensity of the Raman peaks at around 2170 cm^−1^ for Ir^0^/GDY decreased gradually until nearly disappeared, and the G band shifted to lower wavenumbers, which revealed the transition of alkyne bonds to alkene bonds due to the transfer of their π electrons to the adsorbed intermediates. Such changes are completely reversible upon the desorption of the products during catalysis, confirming the reversible ‘alkyne-alkene’ transition of the alkyne-alkene complex [53]. Interestingly, XPS data (Fig. 4h) showed that, during the electro-oxidizing styrene process, the Ir was increased with Ir^3+^. After the reaction, Ir was recovered to Ir of zero valence. This can be understood as follows: at the beginning of the electro-oxidizing styrene process, ST molecules should firstly be adsorbed onto the surface of Ir atoms in Ir^0^/GDY, accompanied with the charge transfer from Ir to ST for the subsequent ST activation, which accordingly leads to the increase of Ir atoms to higher valence states (Ir^3+^). While after the completion of the reaction, all intermediates desorbed from the Ir^0^/GDY catalysts and Ir atoms were recovered to Ir of zero valence (Fig. S31). The obvious incomplete charge transfer between the Ir atoms and GDY guarantees the zerovalent states of the Ir^0^/GDY catalyst.

**Figure 5.**
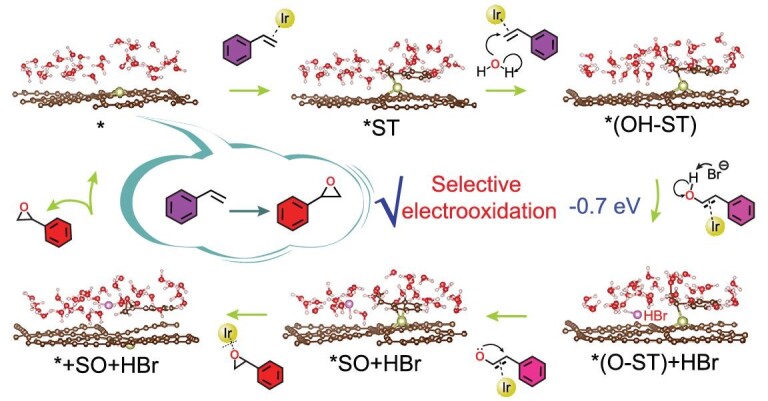
The proposed reaction mechanism of Ir^0^/GDY atomic interface for selective epoxidation reaction.

CDD analysis of *(OH-ST) showed that H atom was electron depletion (Fig. 4i) which could be attacked by Br^−^ easily, and the fractional electron of a total of 0.66 e is loss in H atom. The PDOS of ST-Ir^0^/GDY showed that the electron transformed from Ir 5d orbitals to ST-C 2p orbitals (Fig. 4j), effectively maintaining the absorption and activation of ST. Energy analysis (Fig. 4k; Fig. S32) shows that the reaction on Ir^0^/GDY exhibits mainly a downhill energetic trend with the overall energy gain of −5.72 eV. Accompanied by the DFT calculations, a new reaction mechanism was proposed (Fig. 5) including (i) the activation of ST molecules (*ST, −2.42 eV) by Ir atoms in Ir^0^/GDY, (ii) the formation of the important reactive intermediate, hydroxystyrene (*(OH-ST), −3.57 eV) by the reaction of terminal carbon atoms in *ST with the hydroxyl from water, and (iii) the formation of SO after the attacking of *(OH-ST) by Br^−^ (−0.7 eV). This reaction mechanism is different from those traditionally reported and ensures the highly efficient ST-to-SO conversion.

## CONCLUSIONS

In summary, we have demonstrated an advanced catalytic system of GDY-based zerovalent Ir ACs, comprising of independently and highly monodispersed Ir atom catalysts on GDY, for highly selective and efficient styrene-to-styrene oxides conversion at ambient temperatures and pressures. The experimental results combined with DFT calculations show the intrinsically stable characteristic of Ir^0^/GDY and reveal that the highly selective and active epoxidation of styrene on Ir^0^/GDY proceeds through a distinctive pathway as compared to traditional processes. Our study has demonstrated the real advantages of GDY-based zerovalent ACs in the selective and efficient olefins-to-epoxide conversion process.

## Supplementary Material

nwad156_Supplemental_File
