# Supplementary material for: Ir0/graphdiyne atomic interface for selective epoxidation
Source: Natl Sci Rev. 2023 May 24;10(8):nwad156. doi: 10.1093/nsr/nwad156 (PMC10327882; doi:10.1093/nsr/nwad156)
Supplement: nwad156_Supplemental_File [file nwad156_supplemental_file.pdf]

Supplementary Information for

Ir^0/Graphdiyne Atomic Interface for Selective Epoxidation

Zhiqiang Zheng [1], Lu Qi[1], Yaqi Gao[1], Xiaoyu Luan[1], Yurui Xue[1,*], Feng He[2,3,*] and Yuliang Li[1,2,3*]

[1]Shandong Provincial Key Laboratory for Science of Material Creation and Energy Conversion, Science Center for Ma-terial Creation and Energy Conversion, School of Chemistry and Chemical Engineering, Shandong University, Jinan 250100, China;

[2]CAS Key Laboratory of Organic Solids, Institute of Chemistry, Chinese Academy of Sciences, Beijing 100190, China;

[3]School of Chemical Sciences, University of Chinese Academy of Sciences, Beijing 100049, China.

*Corresponding authors. E-mails: yrxue@sdu.edu.cn; hefeng2018@iccas.ac.cn; ylli@iccas.ac.cn

Contents

Experimental Section **4**

Materials..4

Synthesis of 3D GDY electrodes..4

Synthesis of Ir^0/GDY...4

Morphological and structural characterizations..4

Electrochemical measurements..5

Calculation of the conversion, selectivity and Faradaic efficiency..................................5

XAFS measurements..5

XAFS analysis...5

Calculation setup...6

Supplementary Figures **9**

SEM measurements of GDY...9

Elemental analysis of GDY...10

SEM measurements of CC..11

SEM measurements of Ir^0/GDY...12

Elemental analysis of Ir^0/GDY..13

2D GIWAXS of background...14

TEM measurements of Ir^0/GDY..15

XPS measurements of GDY and Ir^0/GDY...16

Elemental analysis of Ir^0/GDY..17

XAS measurements of Ir^0/GDY..18

First derivative curves of Normalized Ir L_3-edge XANES spectra of Ir^0/GDY.................19

Nyquist plots and Electrochemical active surface area measurement..............................20

Linear sweep voltammetry measurement...21

Tafel plots measurement...22

Mass spectra characterization..23
^{1}H NMR characterization...24
HAADF-STEM measurements of Ir^{0}/GDY...34
XPS measurements of Ir^{0}/GDY..35
Ir valence states analysis...36
Supplementary Tables **37**
Catalytic performance of different catalysts. 37
The conversion and selectivity of Ir^{0}/GDY during the catalytic time.................................38
The yield and Faradic efficiency of Ir^{0}/GDY during the catalytic time.............................39
The variation of the possible products during the catalytic process over Ir^{0}/GDY............40

Experimental Section

Materials. All of the chemicals used were of analytical grade and used directly without any further purification. The carbon cloth was thoroughly cleaned by washed by nitric acid and deionized water before use.

Synthesis of 3D GDY electrodes. The freshly cleaned 3D carbon cloth (3 cm × 4 cm) was added to a 50 mL Teflon-lined stainless-steel autoclave containing 30 mL pyridine solution of hexacetylenebenzene (0.3 mg mL^{-1}). and two pieces of Cu foils. Cu foils act as the catalyst for the growth of GDY nanosheets. The autoclave was then kept at 110 ℃ for 12 h under the protection of Ar. After the completion of the reaction, the 3D GDY electrode was obtained. Prior to uses, the freshly-synthesized 3D GDY electrodes were washed by 3 M HCl, hot DMF, acetone, and 3 M HCl subsequently for at least three times to remove the possible copper residues.

Synthesis of Ir^0/GDY. The zero-valent Ir^0/GDY were synthesized by a facile electrochemical reduction method using a standard three-electrode system, in which the 3D GDY electrode (1 cm × 2 cm), carbon rod and saturated calomel electrode (SCE) were used as the working electrode, counter electrode and reference electrode, respectively. The dilute solution of sulfuric acid (0.5 M) of iridium chloride (5 mM) was used as the electrolyte. During the anchoring of Ir atoms, the GDY electrode was directly immersed in the electrolyte, followed by the in-situ adsorption and reduction of Ir at 10 mA cm^{-2} for 200 s. The obtained Ir^0/GDY was washed by 0.5 M H_2SO_4, and deionized water subsequently for three times, and used for electrocatalysis immediately.

Morphological and structural characterizations. Scanning electron microscope (SEM; Apreo, Thermo Scientific), transmission electron microscope (TEM; Talos F200X G2 TEM, Thermo Scientific), high-resolution TEM (HRTEM) were used to characterize the morphologies of the

samples. Energy-dispersive X-ray spectroscopy (EDX) was collected with an energy-dispersive X-ray detector in Apreo SEM and Talos F200X G2 TEM. HAADF-STEM images were taken from an aberration-corrected cubed FET Titan Cubed Themis G2 300 or JEM-ARM200F (JEOL, Tokyo, Japan). The metal content was determined by inductively coupled plasma optical emission spectrometry (ICP-OES, Agilent ICPOES730). (In-situ) Raman spectra was characterized through the LabRAM HR Evolution spectrometer (the excitation laser source is 473 nm), the sample was scanned with a static raster scan type and a 600 lines per millimeter (L/mm) grating at 50 times magnification. Exposure time of 10 s and two accumulative cycles of 3.2% laser power were used for in situ spectral acquisition. For the in-situ Raman characterization, an electrochemical workstation was attached to the sample cell, the Ir^0/GDY, Pt wire and Ag/AgCl electrode were used as the working electrode, counter electrode and reference electrode, respectively. A mixed solvent of deuterium dimethyl sulfoxide and deuteroxide (3:1, ~0.5 mL) which containing 0.25 M styrene and 0.25 M sodium bromide was used as electrolyte. The in situ electrolysis was conducted at a constant current of 5 mA cm^{-2} for continuous electrocatalysis, the spectrum was collected at 2-minute intervals during the electrocatalysis. X-ray photoelectron spectroscopy (XPS) measurements were carried through a Thermo Scientific Nexsa instrument with monochromatic Al Kα X-ray radiation, the XPS spectra during the electrocatalysis were collected through characterzing the sample with different electrocatalysis time. Nuclear magnetic resonance spectroscopy (^1H NMR, AVANCE NEO) was used to determine the yield and purity of final products. Two-dimensional wide-angle X-ray scattering (2D GIWAXS) experiments were performed at SAXSpoint 5.0 (Anton Paar, Austria).

Electrochemical measurements. The electrochemical measurements were recorded from a CHI 660E electrochemical workstation (Chenhua, Shanghai), equipped with an undivided electrolytic

cell. The Ir^0/GDY or IrNP/GDY, carbon rod and Ag/AgCl electrode were used as the working electrode, counter electrode and reference electrode, respectively. A mixed solvent of 1.5 mL deuterium dimethyl sulfoxide and 0.5 mL deuteroxide which containing 0.5 mmol styrene and 0.5 mmol sodium bromide was used as electrolyte. All potentials were recorded against the Ag/AgCl. Linear sweep voltammetry (LSV) curves are performed to obtain the polarization curves. The galvanostat method was used for electrolysis in corresponding time (0-8h) with a stirring rate of 200 rpm. The chronoamperometry tests were conducted at a constant current of 5 mA cm^{-2} for continuous ST conversion.

Calculation of the conversion, selectivity and Faradaic efficiency. For the oxidation of olefins, the conversion and selectivity were calculated from the ^{1}H NMR results: $Conv. = \frac{S_{St}}{S_{St}+S_{SO}+S_{Br}+S_{OH}}$; $Select. = \frac{S_{SO}}{S_{SO}+S_{Br}+S_{OH}}$.

Where S_{St}, S_{SO}, S_{Br} and S_{OH} are the characteristic peak area of the styrene, styrene oxide, beta-bromostyrene and 1-phenyl-1, 2-ethanediol which integrated from the ^{1}H NMR results.

The Faradaic efficiency was defined from the electric charge consumed for synthesizing styrene oxide and total charge passed through the electrode according to the following equation: $FE_{SO} = \frac{1 \times F \times n_{SO}}{Q}$

Where n_{SO} is the molar number of styrene oxide, F is the Faradaic constant (96485 C mol^{-1}), Q is the total charge passing the electrode.

XAFS measurements. The X-ray absorption find structure spectra were collected at 1W1B station in Beijing Synchrotron Radiation Facility (BSRF). The storage rings of BSRF was operated at 2.5 GeV with an average current of 250 mA. Using Si(111) double-crystal monochromator, the data collection were carried out in transmission/fluorescence mode using ionization chamber. All spectra

were collected in ambient conditions.

XAFS analysis. The acquired EXAFS data were processed according to the standard procedures using the ATHENA module implemented in the IFEFFIT software packages. The L3-weighted EXAFS spectra were obtained by subtracting the post-edge background from the overall absorption and then normalizing with respect to the edge-jump step. Subsequently, L3-weighted $\chi(k)$ data of Ir L-edge were Fourier transformed to real (R) space using a hanning windows (dk=1.0 $Å^{-1}$) to separate the EXAFS contributions from different coordination shells. To obtain the quantitative structural parameters around central atoms, least-squares curve parameter fitting was performed using the ARTEMIS module of IFEFFIT software packages.

The L_3 weighting, and R range of 1.0 – 2.5 Å were used for the fitting. The four parameters, coordination number, bond length, Debye-Waller factor and E0 shift (CN, R, $\sigma2$, ΔE_0) were fitted without anyone was fixed, constrained, or correlated.

Calculation setup. All the density functional theory (DFT) calculations were performed using the Vienna ab initio simulation package (VASP)[1]. To consider the interaction between core electrons and nucleus with valence electrons, the projector augmented plane-wave (PAW) method was employed[2, 3], and the energy cutoff for plane-wave was set to 400 eV. The generalized gradient approximation proposed by Perdew-Burke-Ernzerhof (GGA-PBE) was adopted to consider the exchange-correlation effects[4]. The structure relaxation was finished until the energy was less than 10^{-5} eV and the residual forces on the atoms was less than 0.01 eV/Å. The 3 × 3 × 1 K-points were sampled in the Monhorst-Pack grid for the first Brillouin zone integration. For the electronic structure calculations, the much mesh 5 × 5 × 1 K-points were sampled. To avoid the interaction between periodic repeated slabs, a vacuum layer of 20 Å is added perpendicular to the sheet along

the z-direction. To describe the long-range van der Waals interaction, the DFT-D3 approach was used in the study[5]. During the geometry optimizations, all atoms were allowed to relax. To model the atom catalyst of Ir/GDY, a single layer 2×2 graphdiyne (GDY) supercell with 72 carbon atoms was chosen as the support, and one metal Ir atom was loaded on GDY surface for optimization. The optimized lattice constants of Ir/GDY model are a = b = 18.94 Å with $\alpha = \beta = 90^o$ and $\gamma = 120^o$. For comparison, the graphene supported Ir atom catalyst (Ir/G) with similar size was also built. The differential charge density distribution was calculated using the following formula: $\rho = \rho_{Ir/GDY} - \rho_{GDY} - \rho_{Ir}$, where $\rho_{Ir/GDY}$, ρ_{GDY}, ρ_{Ir} represent the charge density of the whole Ir/GDY, the isolated GDY, and the isolated Ir atom in static calculations, respectively. The charge gain or loss were calculated using the Bader charge method. To consider the solvent effect, the explicit model was employed, in which the Ir/GDY and Ir/G models were surrounded by 32 water molecules using the build block of Materials studio. To examine the dynamic stability of Ir/GDY and Ir/G catalysts, the AIMD simulations were performed using the VASP code[6]. The NVT canonical ensemble methods was employed and the two models were run at 300 K for a period of 10 *ps* with a time step of 1.0 *fs* in the AIMD simulations.

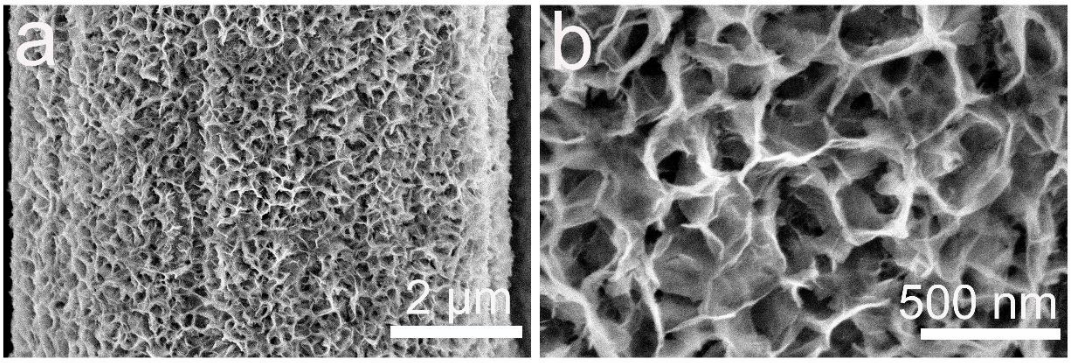

Figure S1. (a) Low- and (b) high-magnification SEM images of GDY.

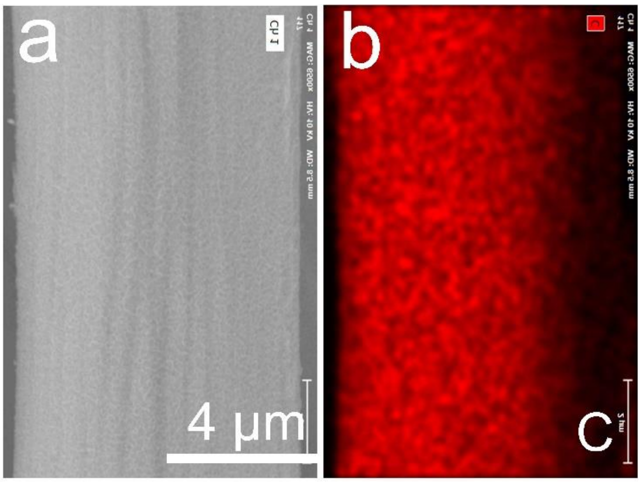

Figure S2. Elemental distribution of GDY.

Elemental mapping analysis shows that only C elements exist in GDY samples.

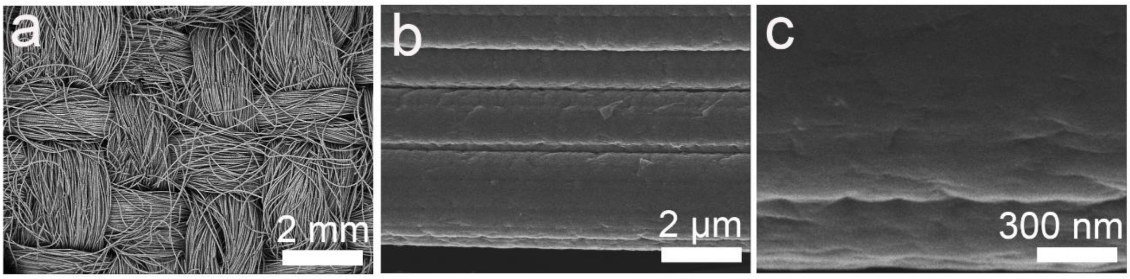

Figure S3. (a) Low- and (b,c) high-magnification SEM images of CC.

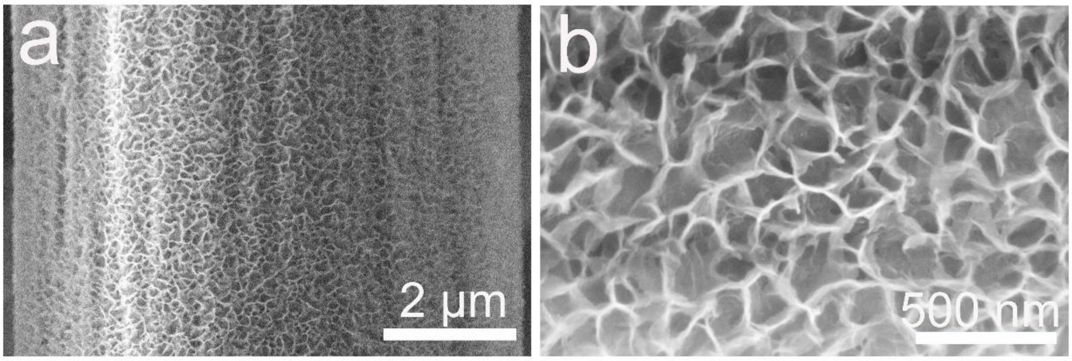

Figure S4. (a) Low- and (b) high-magnification SEM images of Ir^0/GDY.

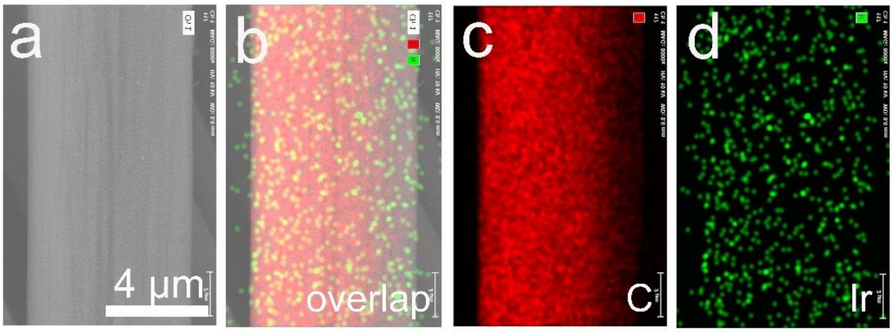

Figure S5. Elemental distribution of Ir^0/GDY.

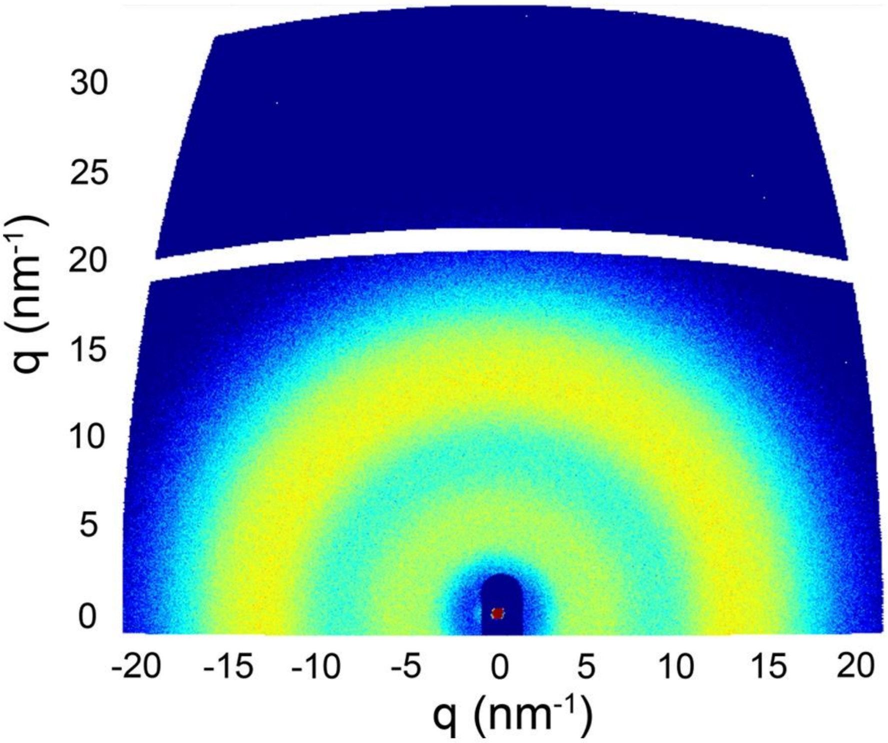

Figure S6. 2D GIWAXS pattern of the background.

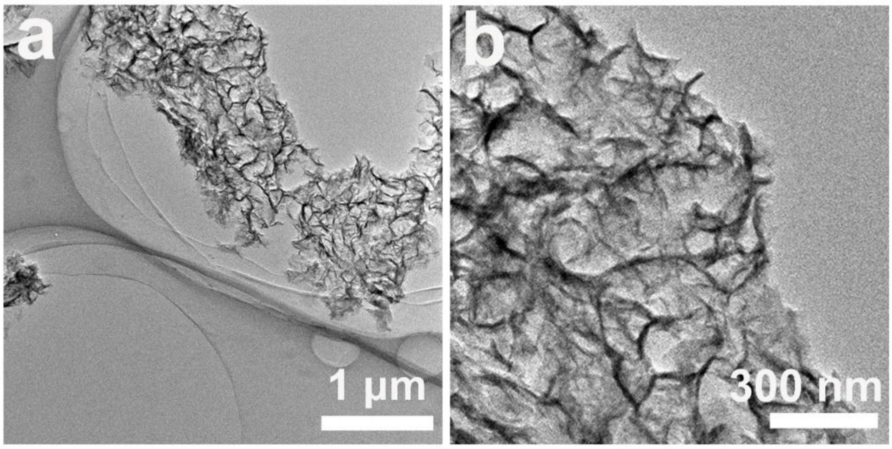

Figure S7. (a) Low- and (b) high-magnification TEM images of Ir^0/GDY.

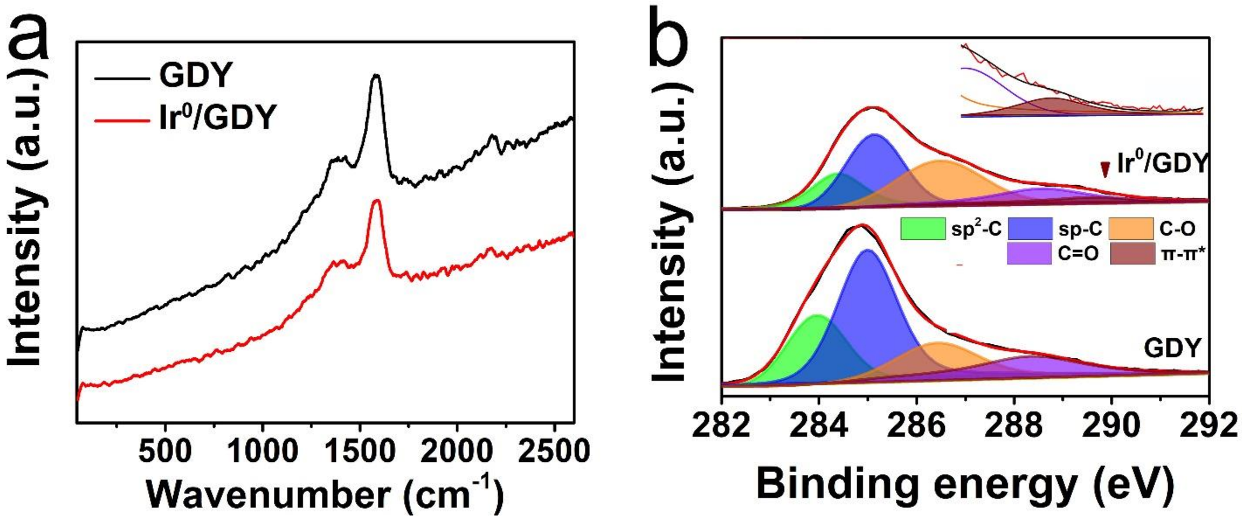

Figure S8. (a) Raman spectra of GDY and Ir^0/GDY. (b) C 1s of XPS spectra of GDY and Ir^0/GDY.

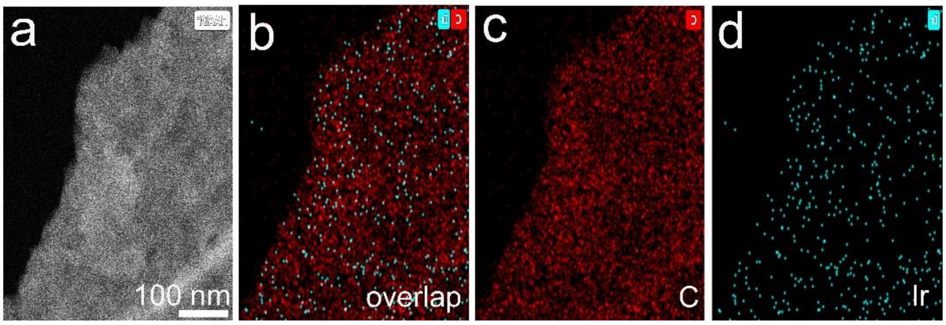

Figure S9. Elemental mapping results of STEM images of C (red) and Ir (blue).

Elemental mapping results demonstrate the uniform distribution of Ir atoms over the GDY nanosheets.

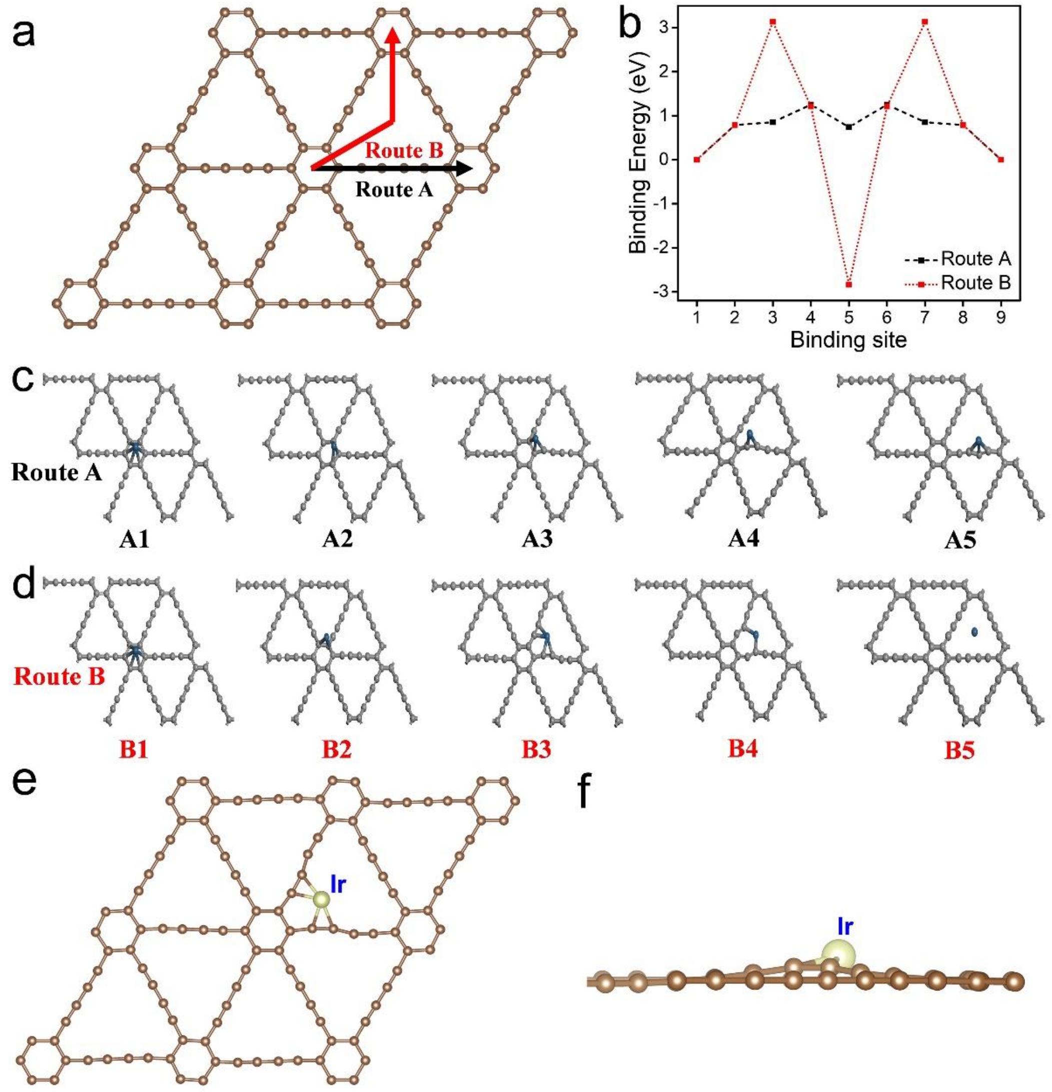

Figure S10. Simulation of the anchored site of the Ir atom on GDY.

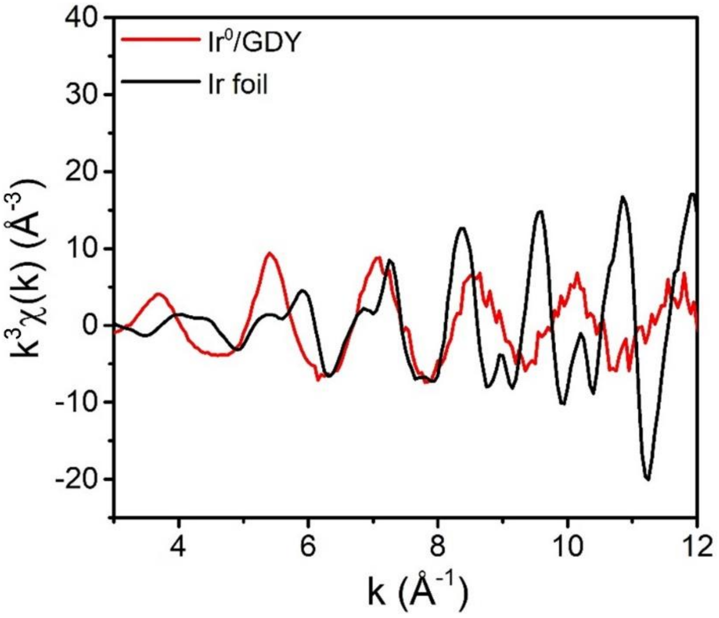

Figure S11. The EXAFS k space curves of Ir^0/GDY (red line) and Ir foil (black line).

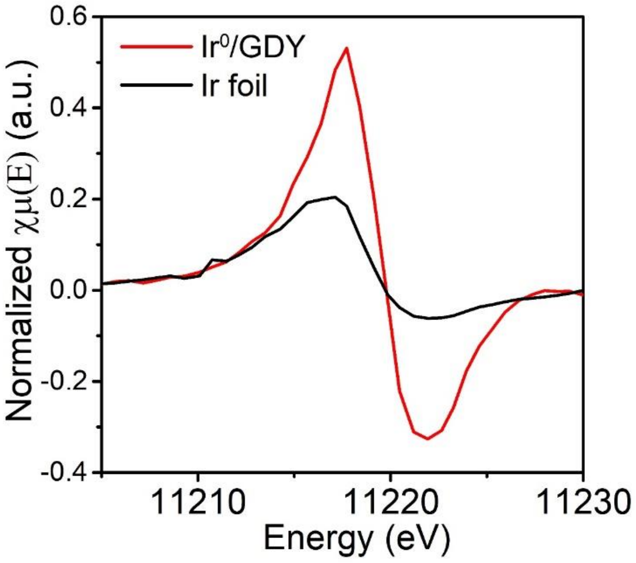

Figure S12. First derivative curves of Normalized Ir L_3-edge XANES spectra of Ir^0/GDY.

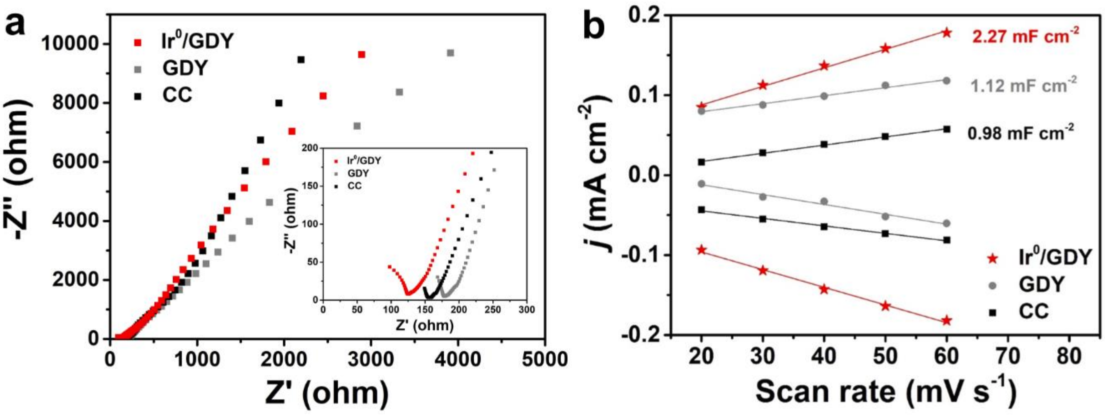

Figure S13. (a) Nyquist plots of EIS and (b) Capacitive current densities plotted with respect to the scan rates for Ir^0/GDY, GDY and CC. the ECSA of Ir^0/GDY, GDY and CC are 56.8 cm^2, 28.0 cm^2 and 24.5 cm^2, respectively.

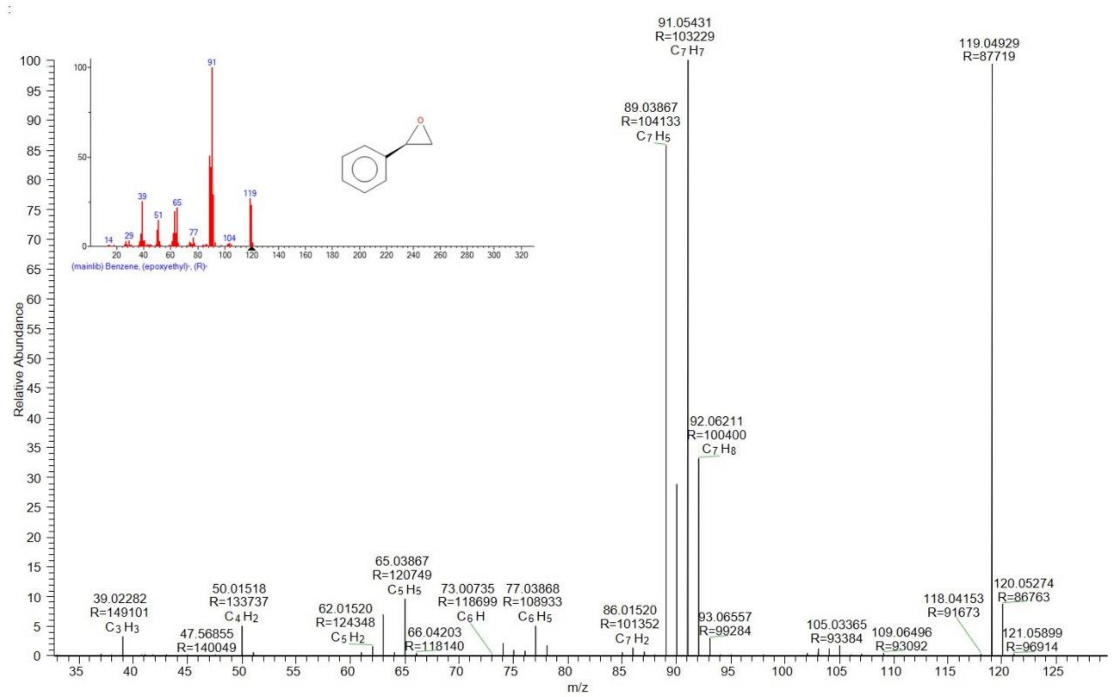

Figure S14. Mass spectra of electrolyte after electrolysis.

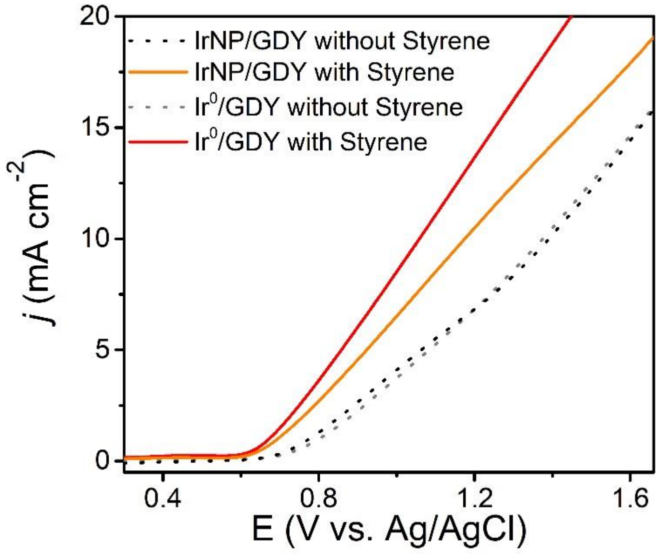

Figure S15. Linear sweep voltammetry (LSV) curves of the sample in the electrolytes with or without ST.

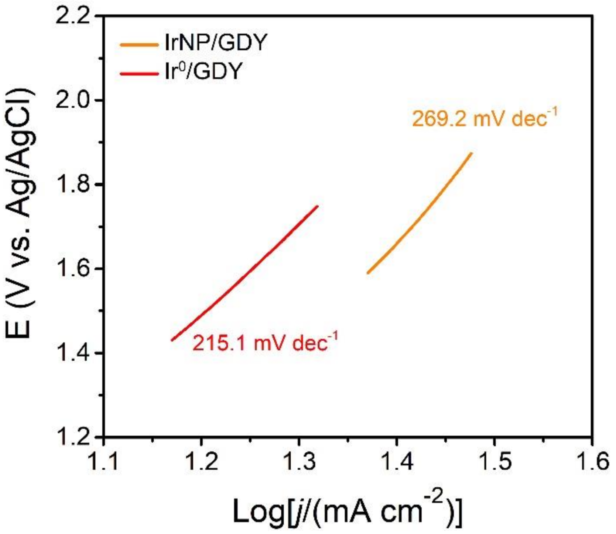

Figure S16. The Tafel plots for IrNP/GDY and Ir^0/GDY.

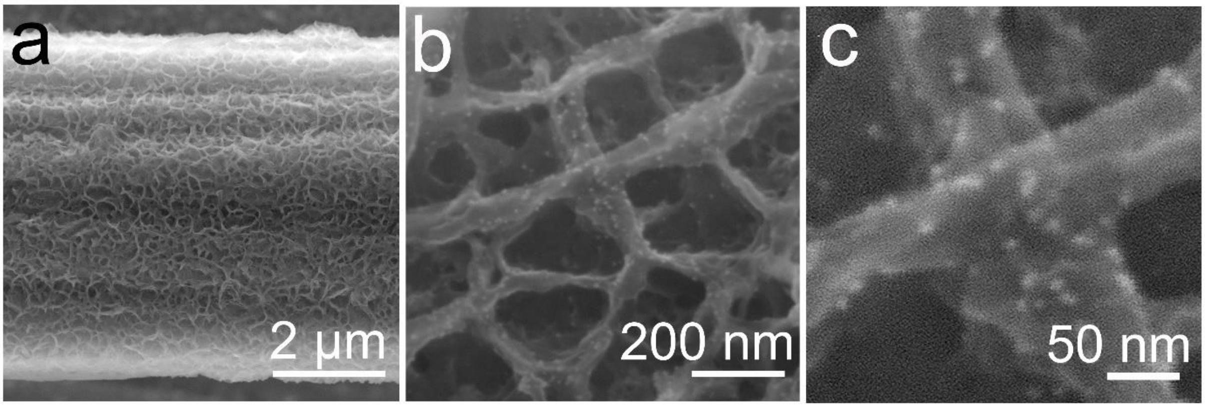

Figure S17. SEM images of Ir-NP/GDY with different magnifications.

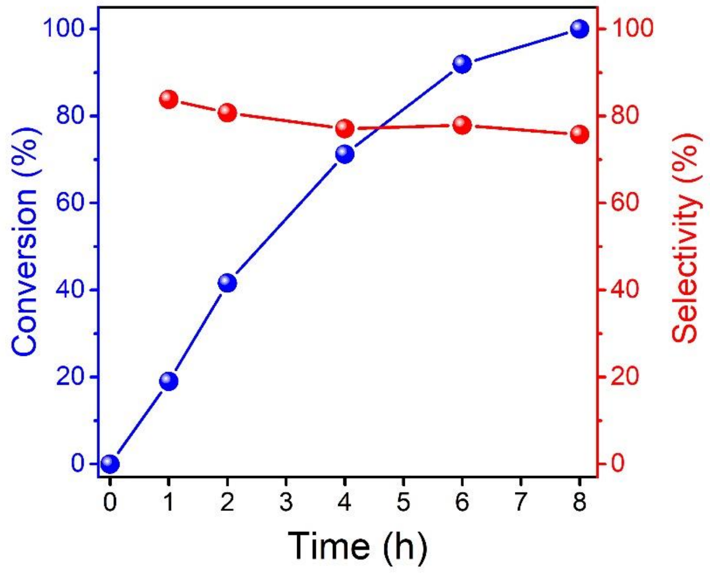

Figure S18. Time course of catalytic performances of Ir-NP/GDY.

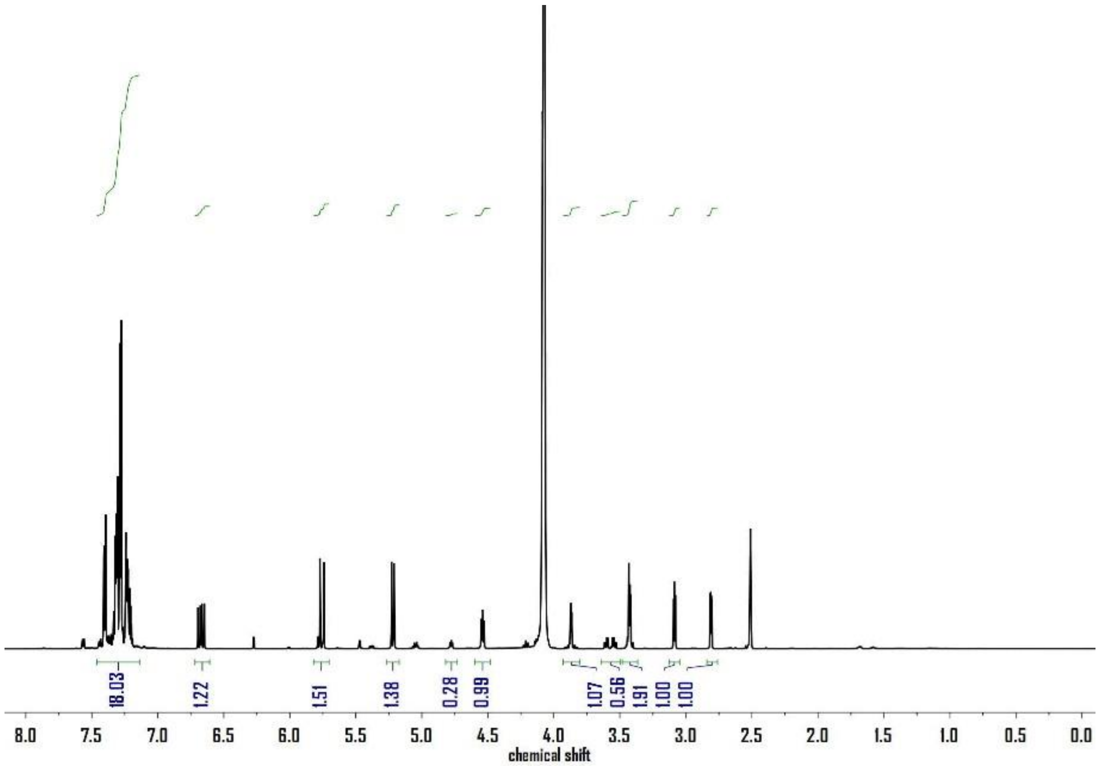

Figure S19. ^{1}H NMR (600 MHz, DMSO-d_6) spectrum of the reaction mixture after the reaction at r.t. for 8 h catalyzed by CC.

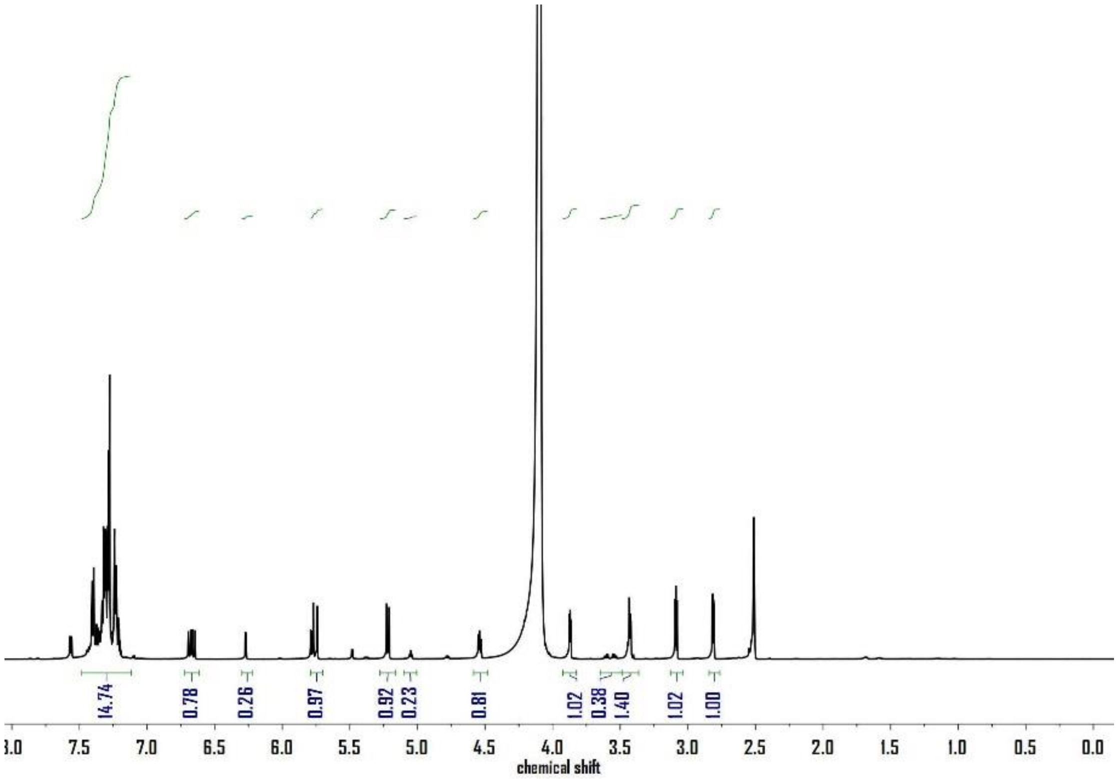

Figure S20. ^{1}H NMR (600 MHz, DMSO-d_6) spectrum of the reaction mixture after the reaction at r.t. for 8 h catalyzed by GDY.

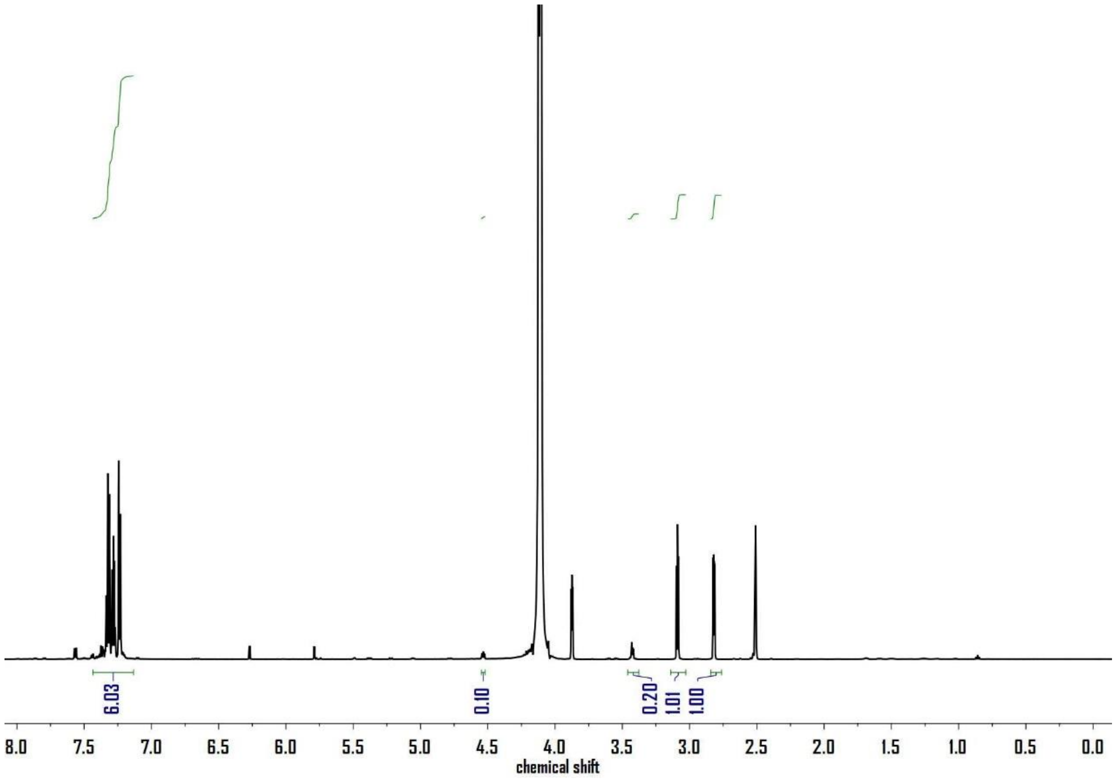

Figure S21. ^1H NMR (600 MHz, DMSO-d_6) spectrum of the reaction mixture after the reaction at r.t. for 8 h catalyzed by Ir^0/GDY.

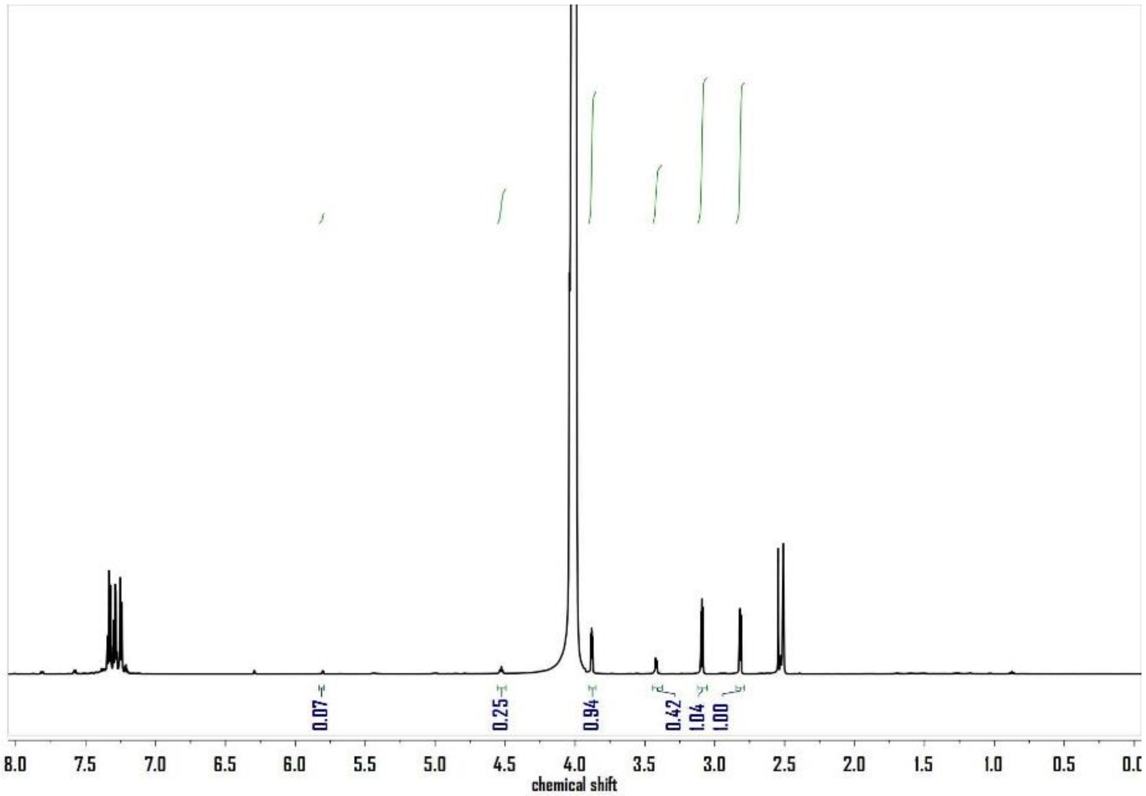

Figure S22. ^{1}H NMR (600 MHz, DMSO-d_6) spectrum of the reaction mixture after the reaction at r.t. for 8 h catalyzed by IrNP/GDY.

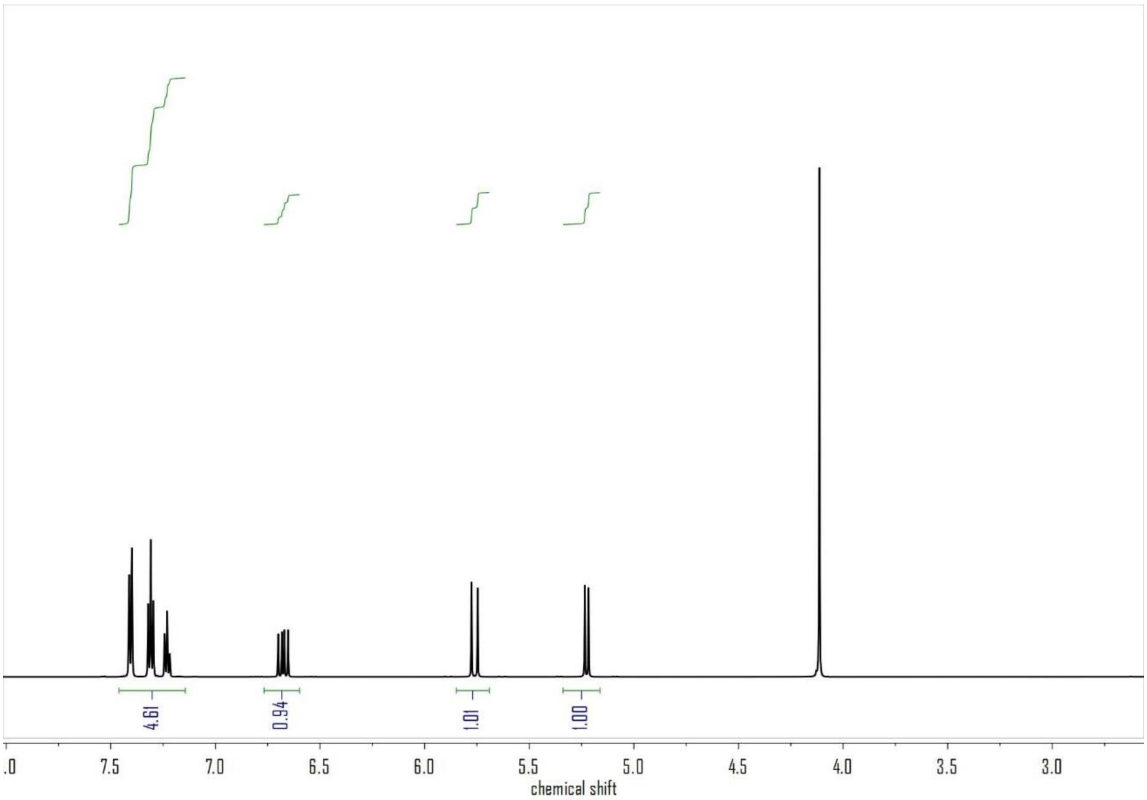

Figure S23. ^{1}H NMR (600 MHz, DMSO-d_6) spectrum of the reaction mixture after the reaction at r.t. for 0 h catalyzed by Ir^0/GDY.

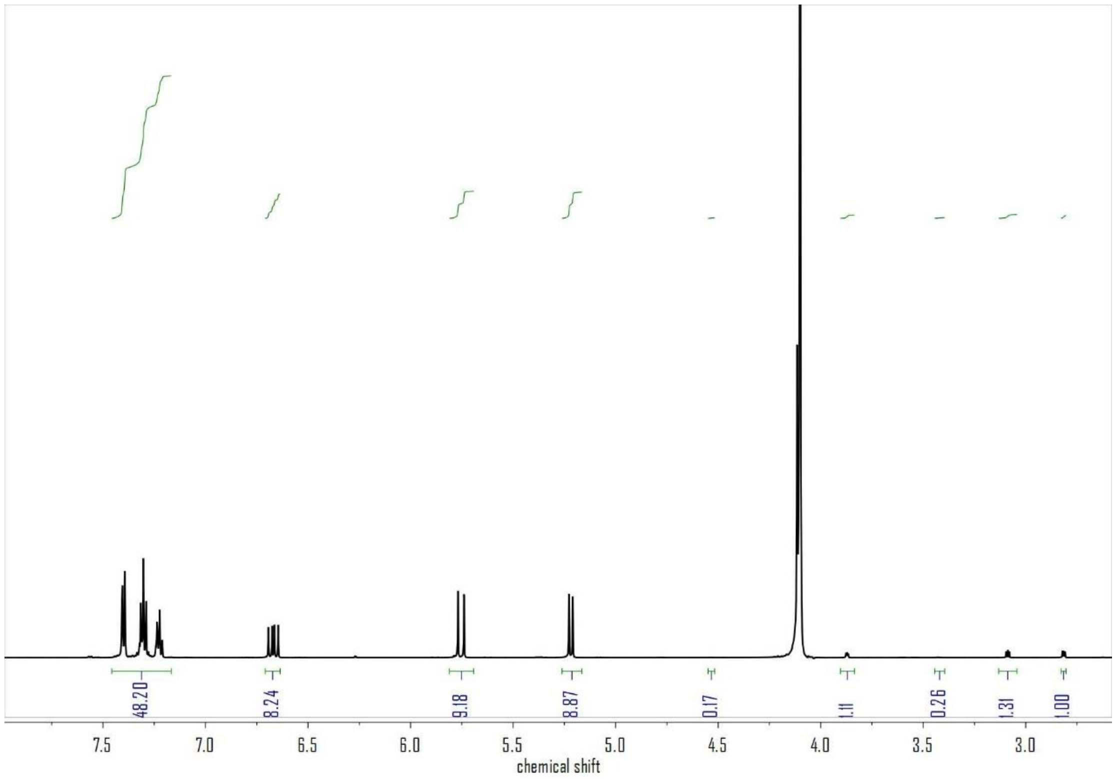

Figure S24. ^{1}H NMR (600 MHz, DMSO-d_6) spectrum of the reaction mixture after the reaction at r.t. for 1 h catalyzed by Ir^0/GDY.

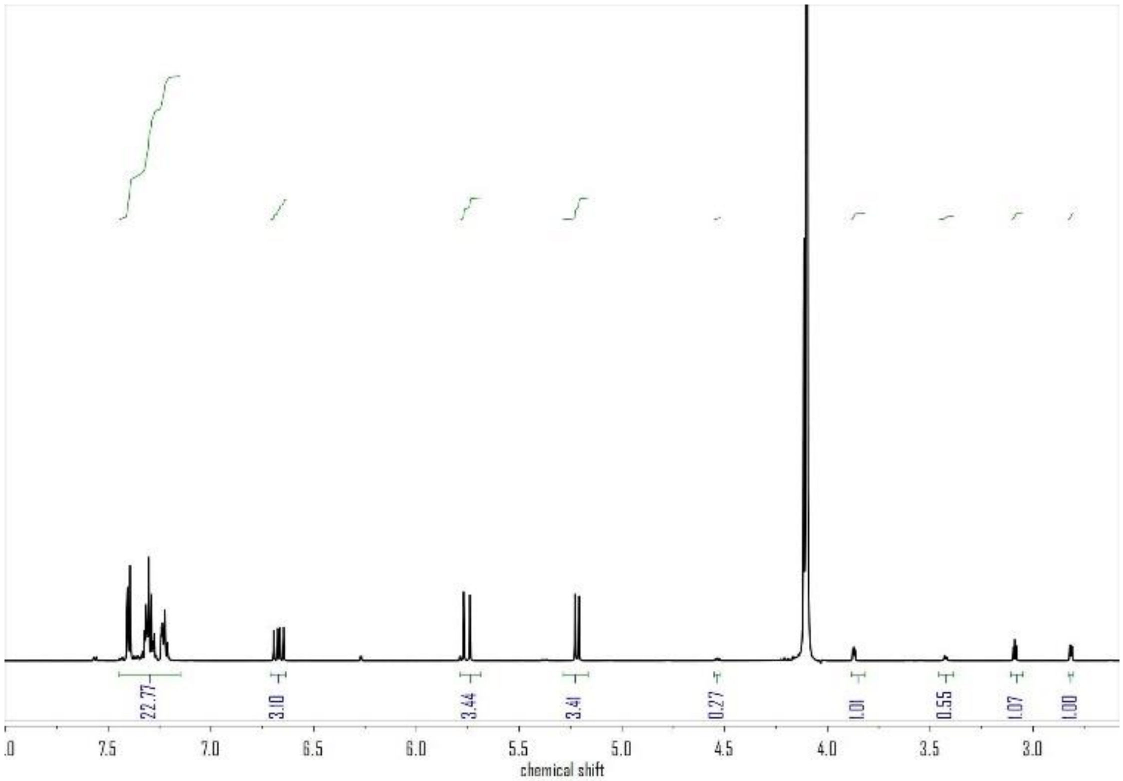

Figure S25. ^{1}H NMR (600 MHz, DMSO-d_6) spectrum of the reaction mixture after the reaction at r.t. for 2 h catalyzed by Ir^0/GDY.

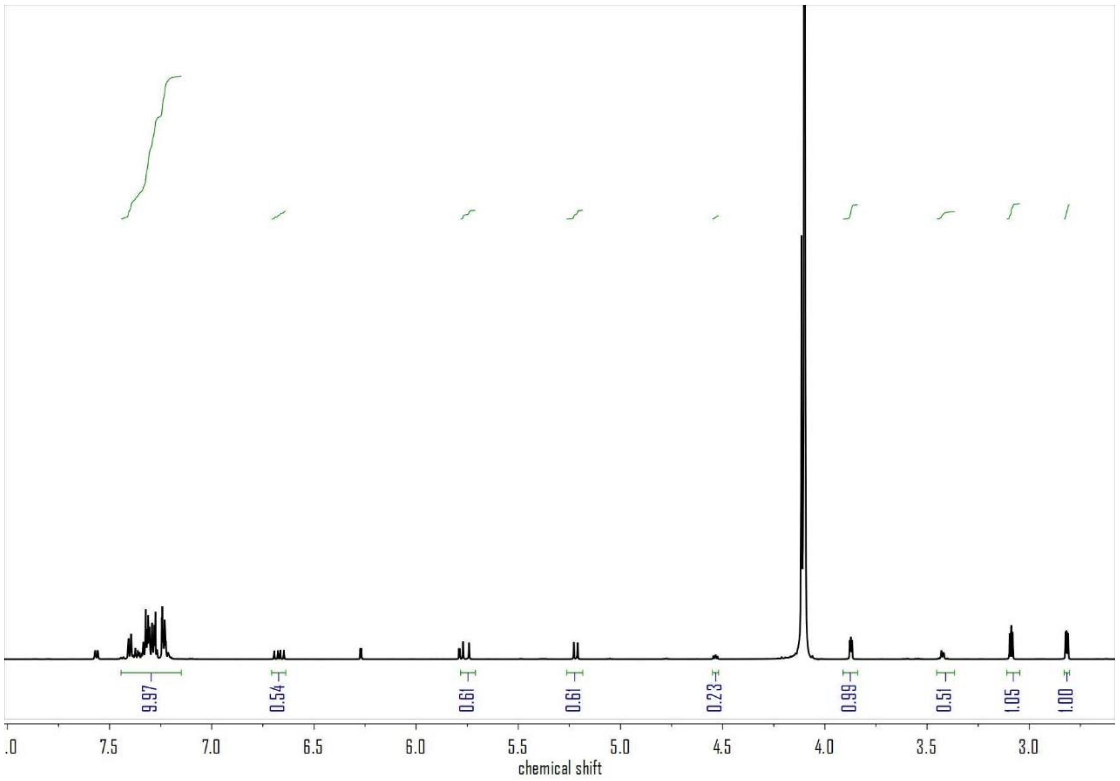

Figure S26. ^{1}H NMR (600 MHz, DMSO-d_6) spectrum of the reaction mixture after the reaction at r.t. for 4 h catalyzed by Ir^0/GDY.

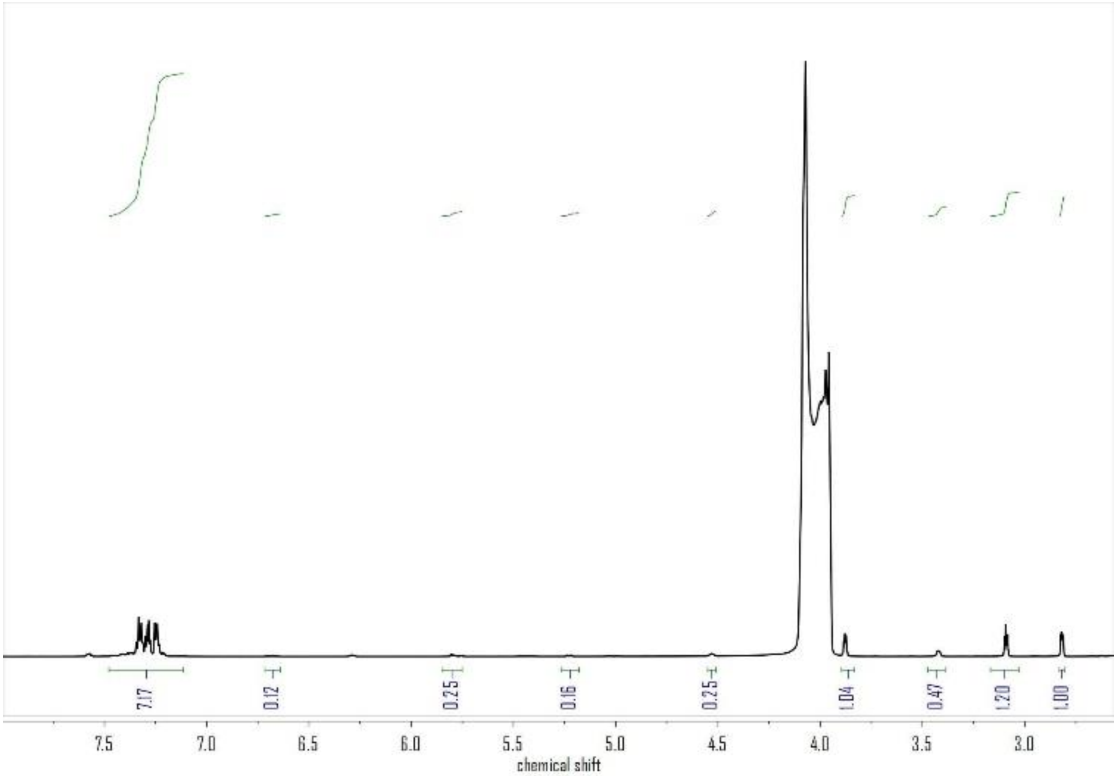

Figure S27. ^{1}H NMR (600 MHz, DMSO-d_6) spectrum of the reaction mixture after the reaction at r.t. for 6 h catalyzed by Ir^0/GDY.

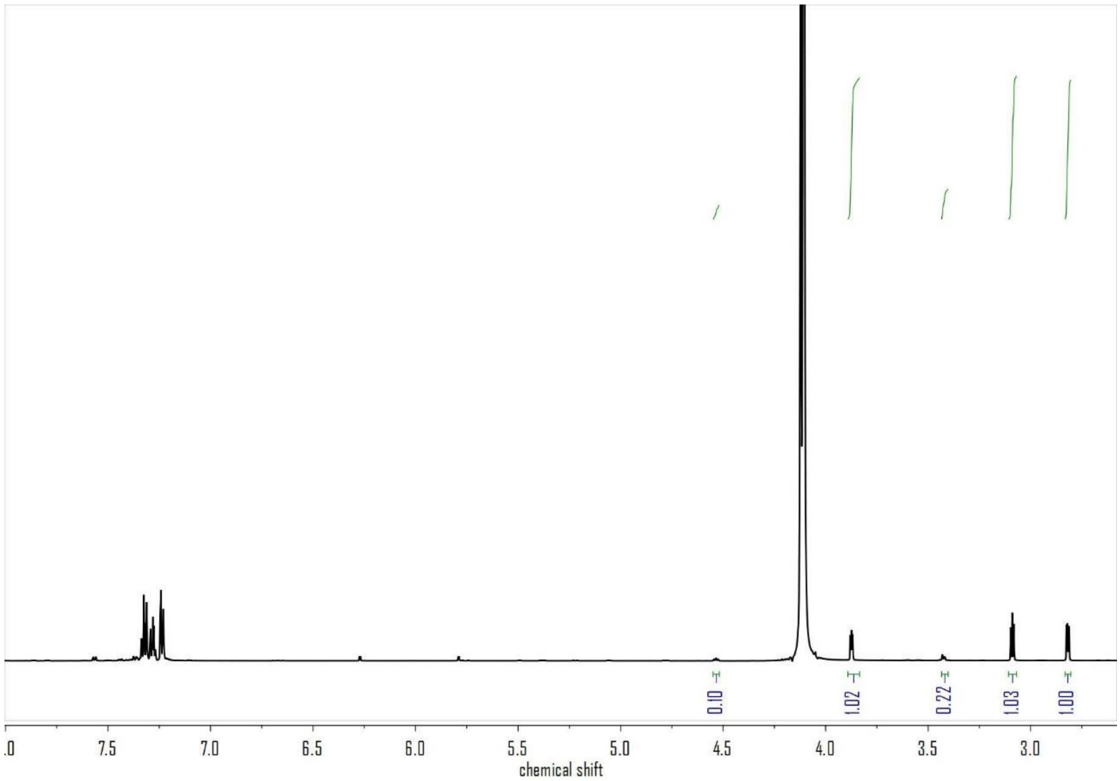

Figure S28. ^{1}H NMR (600 MHz, DMSO-d_6) spectrum of the reaction mixture after the reaction at r.t. for 8 h catalyzed by Ir^0/GDY.

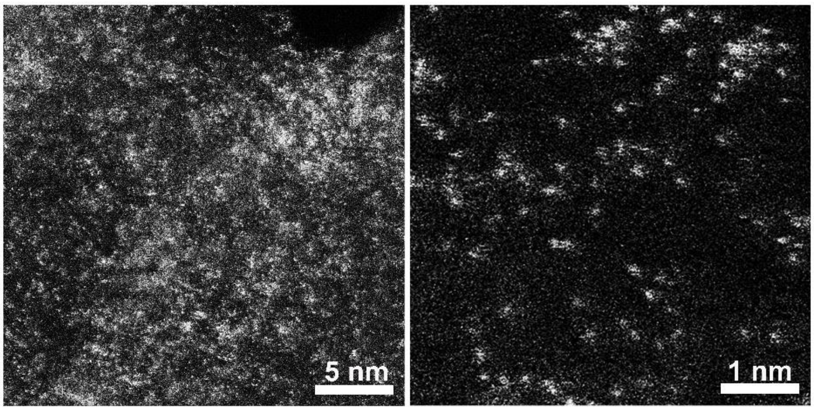

Figure S29. HAADF-STEM images of Ir^0/GDY samples obtained after the electrolysis.

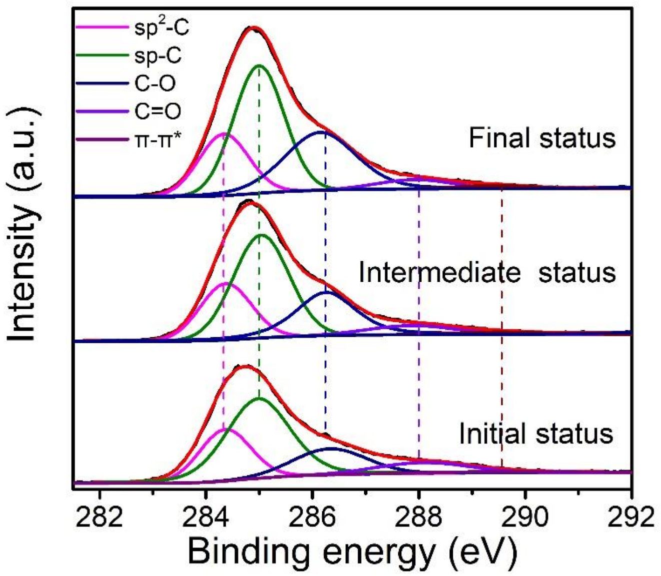

Figure S30. C 1s XPS spectra of Ir^0/GDY recorded during the reaction processes.

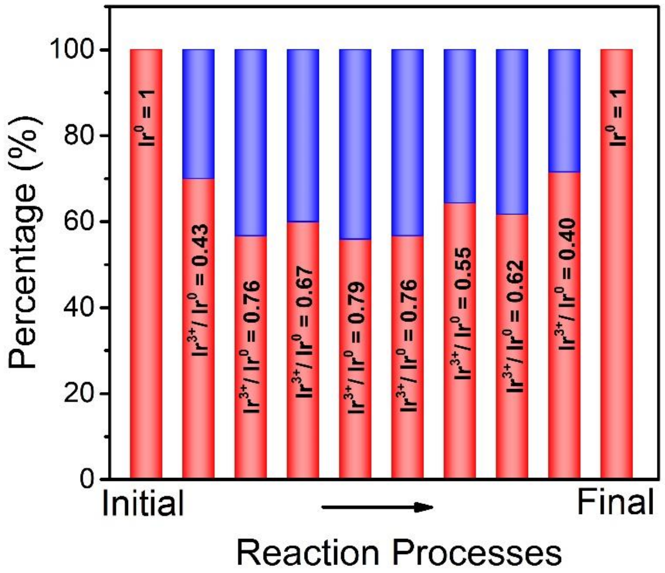

Figure S31. The variation of the Ir valence states during the reaction processes.

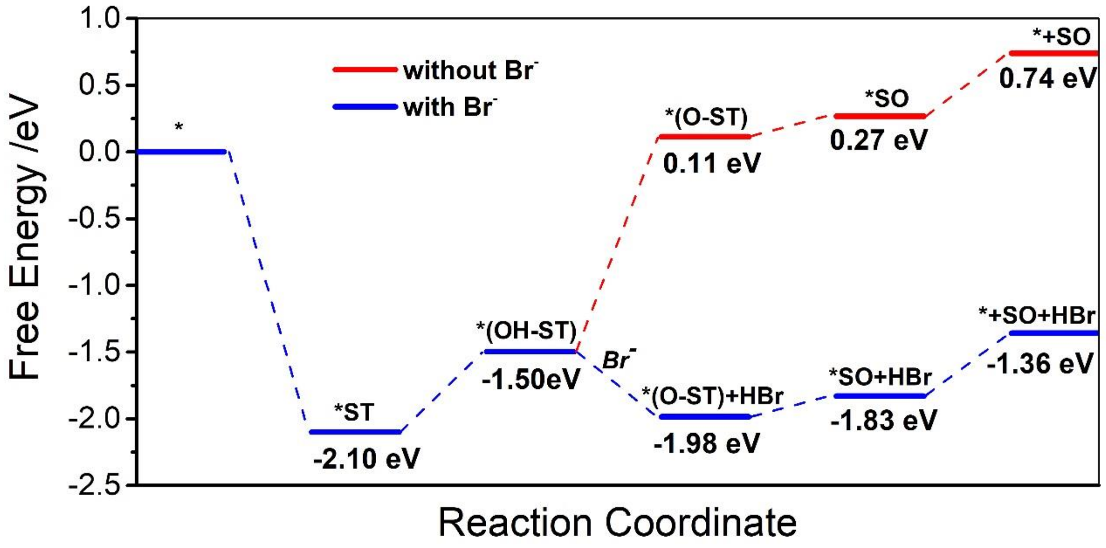

Figure 32. Free energy pathway of the electrocatalysis.

Table S1. EXAFS fitting parameters at the Ir L-edge for various samples

Sample	Shell	N [a]	R (Å) [b]	σ^2 ($Å^2$) [c]	ΔE0 (eV) [d]	*R* factor
1	Ir-C1	2	2.15	0.04	-10.7	*0.025*
	Ir-C2	2	2.53	0.006	-10.7	

[a] *N*: coordination numbers; [b] *R*: bond distance; [c] σ^2: Debye-Waller factors; [d] ΔE_0: the inner potential correction. *R* factor: goodness of fit.

Table S2. Catalytic performance of different catalysts.

Catalysts	Conversion (%)	Selectivity (SO) (%)
**Ir^0/GDY**	**100**	**85.5**
**CC**	**66.5**	**41.3**
**GDY**	**74.3**	**44.3**
**IrNP/GDY**	**100**	**75.8**

Catalytic conditon: 0.25 M ST, 0.25 M NaBr, 2 mL electrolyte, 5 mA cm^{-2}, 8 hour.

Table S3. The conversion and selectivity of Ir^0/GDY during the catalytic time.

**Time (h)**	**Conversion (%)**	**Selectivity (SO) (%)**
**0**	**0**	**--**
**1**	**14.2**	**73.5**
**2**	**32.3**	**67.6**
**4**	**73.3**	**67.6**
**6**	**91.9**	**73.5**
**8**	**100**	**85.5**

Table S4. The yield and Faradic efficiency of Ir^0/GDY during the catalytic time.

**Time (h)**	**TOF (mol g_{Ir}^{-1} h^{-1})**	**Faradic efficiency (%)**
**0**	**0**	**0**
**1**	**64.5**	**46.5**
**2**	**67.6**	**48.8**
**3**	**76.6**	**55.3**
**4**	**69.7**	**50.3**
**5**	**66.1**	**47.7**

Table S5. The variation of the possible products during the catalytic process over Ir^0/GDY.

Time (h)	Styrene (%)	beta-bromostylene (%)	1-phenyl-1,2-ethanediol (%)	Styrene oxide (%)
0	100	0	0	0
1	85.8	2.0	1.8	10.4
2	67.7	4.6	5.9	21.8
4	26.7	12.4	11.4	49.5
6	8.1	7.4	16.9	67.6
8	0	6.0	8.5	85.5

Table S6. The reported catalysts for electro-epoxidation.

Catalyst	Substrate	Time (h)	Current density (mA cm^{-2})	Conv. (%)	Sel. (%)	Faraday efficiency	Reference
Ir^0/GDY	Styrene	8	5	100	85.5	55	This work
Mn_3O_4 NP	Cyclooctene	4	2.5	50	> 65	> 30	J. Am. Chem. Soc. 2019, 141, 6413−6418
CoO NP	Ethylene	-	0.98-7.9	-	-	6.4-17.47	ACS Catal. 2020, 10, 14015−14023
Pt foil	Ethylene	-	300	-	~97	~71	Science 2020, 368, 1228–1233
